# Clinical experiences with the use of oxytocin injection by healthcare providers in a southwestern state of Nigeria: A cross-sectional study

**DOI:** 10.1371/journal.pone.0208367

**Published:** 2019-10-10

**Authors:** Chioma Stella Ejekam, Ifeoma Peace Okafor, Chimezie Anyakora, Ebenezer A. Ozomata, Kehinde Okunade, Sofela Ezekiel Oridota, Jude Nwokike

**Affiliations:** 1 Department of Community Health, Lagos University Teaching Hospital, Lagos, Nigeria; 2 Department of Community Health and Primary care, College of Medicine, University of Lagos, Akoka Lagos, Nigeria; 3 Promoting the Quality of Medicines Program, U.S. Pharmacopeial Convention, Rockville, Maryland, United States of America; 4 Department of Obstetrics and Gynaecology, Lagos University Teaching Hospital, Lagos, Nigeria; Leiden University Medical Center, NETHERLANDS

## Abstract

**Background:**

Postpartum hemorrhage (PPH) is a leading cause of maternal mortality in Nigeria and in most low- and middle-income countries. The World Health Organization (WHO) strongly recommends oxytocin as effective, affordable, and the safest drug of first choice in the prevention and treatment of PPH in the third stage of labor. However, there are concerns about its quality. Very high prevalence of poor-quality oxytocin, especially in Africa and Asia, has been reported in literature. Excessive and inappropriate use of oxytocin is also common in low-resource settings.

**Objective:**

To assess clinical experiences with quality of oxytocin used by healthcare providers in Lagos State, Nigeria.

**Methods:**

This was a descriptive cross-sectional study conducted in 2017, with 705 respondents (doctors and nurses) who use oxytocin for obstetrics and gynecological services recruited from 195 health facilities (public and registered private) across Lagos State. Data collection was quantitative, using a pretested self-administered questionnaire. Data analysis was performed with IBM SPSS version 21. Statistical significance was set at 5 percent (*p*<0.05). Ethical approval was obtained from Lagos University Teaching Hospital Health Research Ethics Committee.

**Results:**

Only 52 percent of the respondents knew oxytocin should be stored at 2°C to 8°C. About 80 percent of respondents used oxytocin for augmentation of labor, 68 percent for induction of labor, 51 percent for stimulation of labor, and 78 percent for management of PPH. Forty-one percent used 20IU and as much as 10% used 30IU to 60IU for management of PPH. About 13 percent of respondents reported believing they had used an ineffective brand of oxytocin in their practice. Just over a third (36%) had an available means of documenting or reporting perceived ineffectiveness of drugs in their facility; of these, only about 12 percent had pharmacovigilance forms in their facilities to report the ineffectiveness.

**Conclusion:**

The inappropriate and inconsistent use of oxytocin, especially overdosing, likely led to the high perception of medicine effectiveness among respondents. This is coupled with lack of suspicion of medicine ineffectiveness by clinicians as a possible root cause of poor treatment response or disease progression. Poor knowledge of oxytocin storage and consequent poor storage practices could have contributed to the ineffectiveness reported by some respondents. It is necessary to establish a unified protocol for oxytocin use that is strictly complied with. Continuous training of healthcare providers in medicine safety monitoring is advocated.

## Background

Poor maternal and child health indices have remained a recurring public health challenge in Nigeria. Obstetric hemorrhage, especially postpartum hemorrhage (PPH), is a leading cause of maternal mortality in Nigeria [1,2]. According to the World Health Organization (WHO), PPH is defined as a blood loss of 500 mL or more within 24 hours after birth [2]. It is said to affect approximately 2 percent of all women who give birth and is associated with nearly one-quarter of all maternal deaths globally [2]. In 2015 Nigeria and India accounted for approximately 58,000 maternal deaths, more than one-third of global maternal deaths [3]. Fortunately, deaths from PPH are preventable. WHO strongly recommends oxytocin as effective, affordable, and the safest drug of choice in the prevention and treatment of PPH [2]. Oxytocin is also used intrapartum for induction, stimulation, and augmentation of labor when medically indicated and where benefit outweighs risk [4,5]. The UN Commission names oxytocin 1 of the 13 lifesaving commodities within the continuum of care to effectively address avoidable causes of death during pregnancy and childbirth[6] and WHO includes oxytocin in the WHO Model List of Essential Medicines[7].

However, there are concerns about the quality of available oxytocin. To maintain its quality, oxytocin requires stable cold chain storage from the point of manufacture to the point of use [8]. It is recommended to be stored in the refrigerator at 2°C to 8°C[8]. A major problem with oxytocin relates to heat-related degradation due to inappropriate storage in the supply chain and at health facilities. In most low-income countries, these storage conditions are usually very difficult to maintain [9,10]. Surveillance studies have shown high prevalence of poor-quality oxytocin, particularly in Africa and Asia.[10–12] Most common problems included insufficient amounts of or no active ingredient [10,11].

Safe medicines supply is fundamental to public health, and poor-quality medicines have the greatest potential for harming the health of consumers, with far-reaching consequences, including treatment failure, adverse drug reactions, economic hardship, health problems, and death [13]. Poor-quality uterotonics have dire consequences: apart from increased maternal mortality, they can lead to performing surgical procedures that could have been prevented [14].

In a recent study in Nigeria, the quality audit of oxytocin injections in circulation showed an alarming failure rate: up to 74 percent of sampled oxytocin injection failed the quality test [10]. Despite this evidence and concerns around poor-quality medicines, epidemiologic data around quality of medicines are still sparse and poor. Many healthcare providers do not generally suspect the medicines they are using as a cause of disease progression and contributor to treatment outcome. Reports have it that obstetricians in sub-Saharan Africa often give three vials of oxytocin to ensure they get the equivalent of at least one dose, as prevention of PPH with one vial of oxytocin is difficult [14]. Reports also suggest that knowledge of proper oxytocin storage may be inadequate. A previous study reported poor knowledge of healthcare providers regarding safe storage of oxytocin [15]. This current study serves as a sequel to the quality audit of oxytocin injections in Nigeria and seeks to assess the clinical experience of healthcare providers in Lagos State, Nigeria, with the quality of oxytocin injection used. It tries to assess what healthcare providers know about oxytocin injection, how they use it, their clinical experiences with use, and their perceptions of the effectiveness of the medicines. In this study, *effectiveness* is defined as the ability of the oxytocin injection used to achieve the desired contraction within the recommended dose for a specific indication [16].

## Materials and methods

### Study population

A descriptive cross-sectional study was conducted to assess the clinical experiences of healthcare providers in Lagos State with oxytocin use. The study population consisted of practicing doctors and nurses working in either public or private facilities in Lagos State. To participate in the study, respondents had to be employed in registered public or private health facilities in Lagos State that offered obstetrics and gynecological services and use oxytocin in their practice.

### Sample size determination

The sample size was determined using Cochrane’s formula, considering the following criteria: a standard normal deviation with 95% confidence, with 5% accepted error of margin and proportion of reported effectiveness (52.5%) of another uterotonic from a previous study in Nigeria[17].

$$n=\frac{z^{2}pq}{e^{2}}$$

Where: *n* = minimum required sample size, *z* = standard normal deviation (95% confidence = 1.96), *e* = accepted error of margin (5%), *p* = proportion of reported effectiveness of misoprostol from a previous study in Nigeria (52.5%), and

*q* = 1−*p*.

Thereby:

$$n=\frac{{\left(1.96\right)}^{2}(0.525)(0.475)}{{\left(0.05\right)}^{2}}$$

n=384

Providing for a 30-percent nonresponse rate (to a self-administered questionnaire), the minimum calculated sample size will be 499.

### Sampling technique and selection of respondents

Multistage sampling was used to select public and private healthcare facilities from each of the five administrative divisions in Lagos State. Multistage sampling was chosen in order to divide the State into preferred smaller units of administrative divisions to guide the selection of respondents and ensure representativeness.

### Stage 1: Selection of Local Government Areas (LGAs) from the five administrative divisions

A simple random sampling with ballot paper was used to select four LGAs from Ikeja administrative division, two from Badagry, two from Lagos Island, one from Epe, and one from Ikorodu. This amounted to 10 LGAs from a total of 20 LGAs in Lagos State.

### Stage 2: Selection of public and private facilities

The three tertiary health facilities that provide obstetrics and gynecological services in Lagos State were purposively selected. Every secondary level public healthcare facility/general hospital and comprehensive primary healthcare center (PHC) in the selected LGAs was included for recruiting respondents from the public health sector.

### Stage 3: Selection of the private health facilities

Using the list of registered private hospitals per LGA as provided by the Lagos State Ministry of Health, 15 private healthcare facilities that offer obstetrics and gynecology services were selected by systematic sampling per LGA. This came to a total of 150 private health facilities from the 10 LGAs.

### Stage 4: Selection of healthcare providers

To ensure representativeness, based on the proportion of doctors to nurses in the public and private sectors according to the Human Resources for Health indices in Lagos State, 60 percent of respondents selected for the study were from the private health sector, and 40 percent were from the public health sector. A doctor-to-nurse ratio of 1:2 was used to select respondents who met the inclusion criteria and signed the informed consent form confirming willingness to participate from both the public and private health facilities.

Overall, 705 respondents (doctors and nurses) who use oxytocin were recruited from 195 health facilities, including 3 public tertiary facilities, 10 general hospitals; 32 comprehensive PHCs, and 150 private health facilities across the 5 administrative divisions of Lagos State. This is shown in Table 1.

**Table 1:** Sampling technique.

Stage of sampling	Activity	No. of samples
Stage 1	Selection of LGAs from the 5 administrative divisions in Lagos State	10 LGAs selected
Stage 2	Selection of public health facilities	3 tertiary health facilities 10 general hospitals 32 comprehensive PHCs
Stage 3	Selection of private health facilities	150 private health facilities
Stage 4	Selection of healthcare providers	Doctor-to-nurse ratio per facility was 1:2

Table 1 gives a description of every stage of the sampling technique.

### Data collection technique and management

Quantitative data were collected using a pretested self-administered questionnaire, which was developed following a literature review and incorporated expert contributions, reviews, and opinions. The questionnaire was pretested among 20 healthcare providers (doctors and nurses) who met the inclusion criteria and were from facilities in the LGAs not selected for this study. The questionnaire sought information on sociodemographic and occupational history of respondents, general obstetric knowledge, and clinical experience with oxytocin use. The main outcome measures were the proportion of healthcare providers with good knowledge of oxytocin storage, pattern of oxytocin usage and dosing by healthcare providers, proportion of oxytocin perceived to be effective and ineffective by healthcare providers, and proportion of healthcare providers who document or take action concerning perceived ineffectiveness of oxytocin in their clinical practice. Data entry, cleaning, and analysis were performed using IBM SPSS v.21. The variables were discrete quantitative variables. Data were presented in frequency tables using simple proportions. The mean and standard deviation were used to summarize quantitative variables that were normally distributed, while median and interquartile ranges (IQRs) were used for those that were found not to be normally distributed. Inferential statistics were done with chi-square tests, and statistical significance was set at *p*<0.05. Since the responses in the study were self-reported, the possibility of recall bias and social desirability bias existed, but setting the recall period to the past 1 year would minimize recall bias. Many options were offered on each question for respondents to choose from, and the questionnaire was self-administered without respondent’s personal details (anonymity), which reduced the chances of social desirability bias. In addition, respondents were required to complete the questionnaires and return them immediately, as they took about 10 minutes to complete. The questionnaire clearly stated there were no right and wrong answers and that the study only sought to assess current practice. Because it was a self-administered questionnaire, there was the possibility of none-response bias, which was minimized by almost doubling the minimum calculated sample size. Ethical approval was obtained from the Health Research and Ethics Committee of the Lagos University Teaching Hospital, Lagos Nigeria (HREC assigned no. ADM/DCST/HREC/APP/1800). Formal consent was obtained from each respondent.

## Results

A total of 705 respondents participated in the study. Table 2 shows the sociodemographic characteristics of the respondents. They were mostly within the 30- to 40-year age bracket (41.4%) with a mean age of 36.3±10.4 years. There were more females (71.6%) and nurses (61.0%). More respondents came from the private sector (62.1%). The majority of respondents had worked for 10 years or less (64.5%), with a median of 7.5 years. Nearly all of the respondents (92.9%) had received some form of training on oxytocin.

**Table 2:** Sociodemographic characteristics of respondents (*n* = 705).

Characteristic	Category	Frequency	Percent
Age group (years)*	<20	2	0.3
	20–29	195	27.7
	30–39	292	41.4
	40–49	124	17.6
	50–59	66	9.4
	≥60	26	3.6
Sex	Males	200	28.4
	Females	505	71.6
Years of practice^†^	1–10	455	64.5
	11–20	146	20.7
	21–30	73	10.4
	≥31	31	4.4
Cadre of health worker	Doctor	275	39.0
	Nurse	430	61.0
Sector of practice	Public	267	37.8
	Private	438	62.1
Training on oxytocin use	Yes No	655 50	92.9 7.1

* Mean 36.3, SD ± 10.4

† Median 7.5, IQR (4, 15)

**Fig 1:**
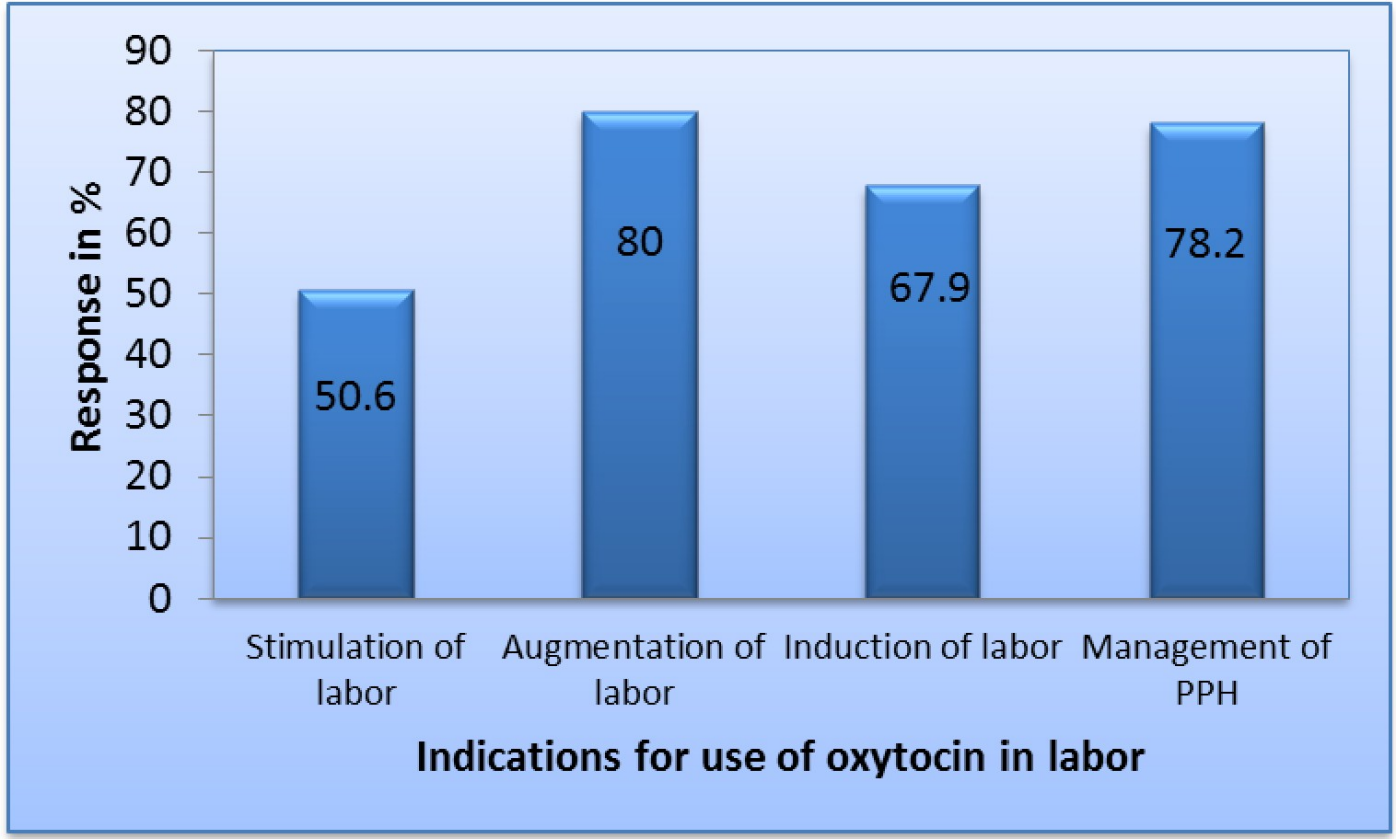
Indications for use of oxytocin among respondents.

Table 3 shows the respondents’ general obstetrics knowledge and practice. Most knew the correct definition of PPH (86.2%). Just over half (52.2%) knew the proper storage place for oxytocin (the refrigerator at 2°C–8°C, while as many as 42 percent stored their oxytocin on shelves. Analysis of the indications for use of oxytocin among respondents showed that 80 percent of respondents used oxytocin for augmentation of labor, 78.2 percent for management of PPH, 68 percent for induction of labor, and 50.6 percent for stimulation of labor, as shown in Fig 1.

**Table 3:** General obstetrics knowledge and practice of the respondents (n = 705).

Aspect	Question	Frequency (%)
Knowledge of PPH	Correct definition (Yes)	608 (86.2)
Respondents use of oxytocin in obstetrics	Stimulation of labor	357 (50.6)
	Augmentation of labor	564 (80.0)
	Induction of labor	479 (67.9)
	Management of PPH	551 (78.2)
Storage of oxytocin	Fridge	368 (52.2)
	Shelf	297 (42.1)
	Dark	30 (4.3)
	Others	10 (1.4)

**Fig 2:**
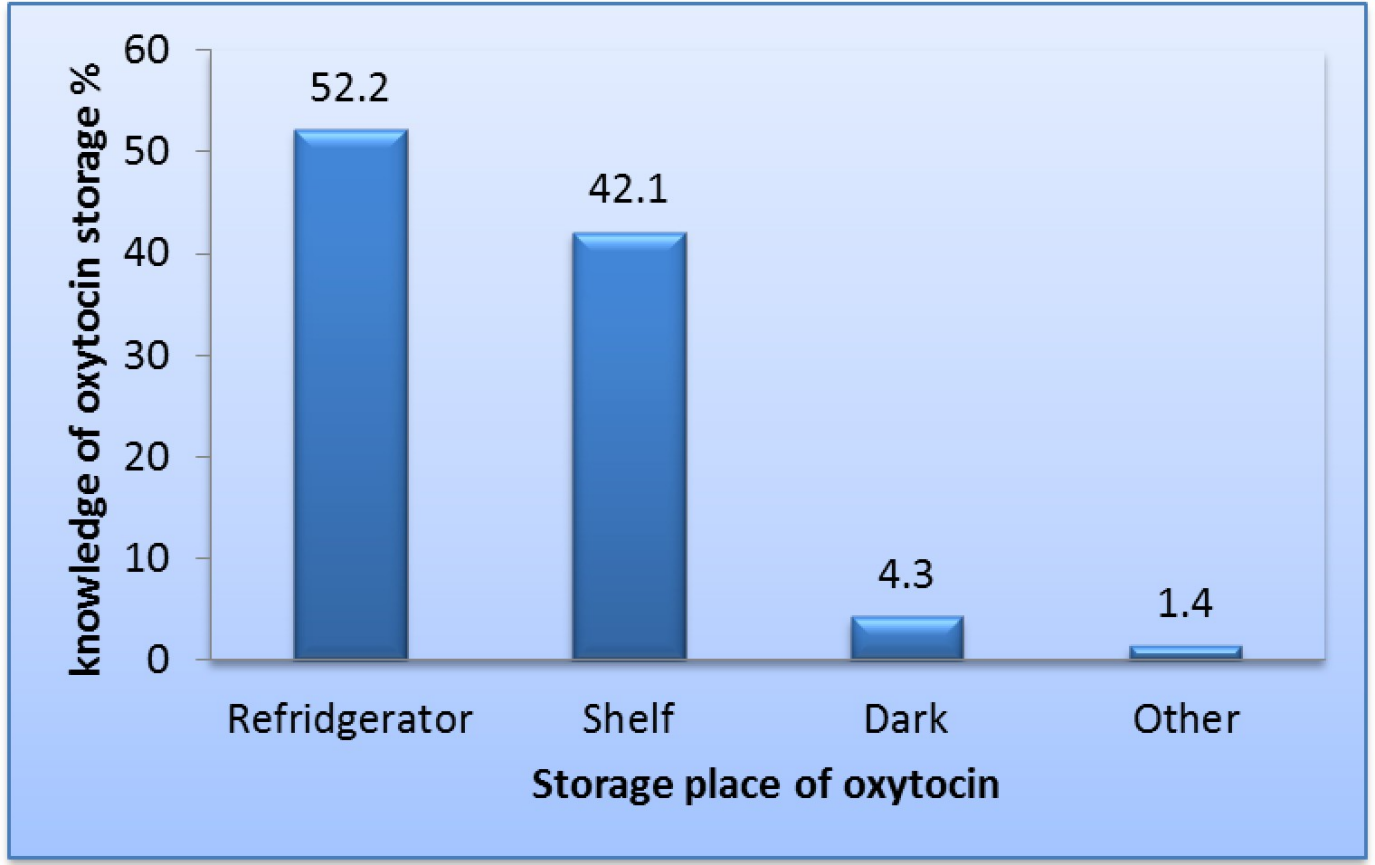
Knowledge of oxytocin storage.

Table 3 presents respondents’ knowledge of definition of PPH, knowledge of the recommended storage for oxytocin, and what they use oxytocin for. More doctors (59.3%) than nurses (47.7%) knew that oxytocin should be stored in the refrigerator, and more respondents in government facilities (68.4%) than in private facilities (40.2%) knew the proper storage for oxytocin (Table 4). Table 4 also presents the analysis of the assessment of respondents’ knowledge of the recommended storage place for oxytocin by healthcare provider cadre and by sector of practice. Fig 2 shows respondents’ understanding of the proper storage conditions.

**Table 4:** Assessment of knowledge of oxytocin storage by Cadre and Sector of practice of respondents.

Variable	Frequency, *n* = 705(%)	
**Storage of oxytocin**	**Doctors, *n* = 275 (%)**	**Nurses, *n* = 430 (%)**
Refrigerator	163 (59.3)	205 (47.7)
Shelves	93 (33.8)	204 (47.4)
Dark	14 (5.1)	16 (3.7)
Others	5 (1.8)	5 (1.2)
**Storage of oxytocin**	**Public, *n* = 267 (%)**	**Private, *n* = 438 (%)**
Refrigerator	182 (68.4)	176 (40.2)
Shelves	66 (24.8)	217 (49.5)
Dark place	3 (1.1)	27 (6.2)
Others	16 (6.0)	18 (4.1)

Twenty-three percent of the respondents monitored the effectiveness of oxytocin using the frequency and duration of uterine contraction while 65% used frequency/duration of uterine contractions and cervical dilatation as shown in Table 5.

**Table 5:** Respondents’ general practice with oxytocin (*n* = 705).

Questions	Response	Frequency (%)
Cadre administering oxytocin	Doctors	165 (23.4)
	Nurses	46 (6.6)
	Both	494 (70.0)
Responsibility for procurement of oxytocin	Clients	95 (13.5)
	Facility	610 (86.5)
Indicator for monitoring effectiveness of oxytocin in labor	Correct frequency and duration of uterine contractions	165 (23.4)
	Cervical dilatation	73 (10.4)
	Both of the above	461 (65.4)
	None of the above	3 (0.4)
	Others	3 (0.4)

Table 5 shows the cadre administering oxytocin in respondents’ clinical practices, who procures, and their indicator for monitoring the effectiveness of oxytocin used during labor.

Table 6 summarizes respondents’ indication for use and dosage of oxytocin for various obstetric indications. About 48 percent of respondents indicated they used 10IU of oxytocin for stimulation/augmentation of labor in primigravida, 24 percent used 5IU, and 17 percent used 20IU, while 2.4 percent used other doses ranging from 30IU to 60IU. About 40 percent of respondents used 5IU for stimulation/augmentation of labor in multiparas, 41 percent used 10IU, 10 percent used 20IU, and as many as 4.4 percent used other doses ranging from 30IU and 60IU. Concerning the dosing of oxytocin for preventing PPH, 41.4 percent used 20IU, 4.8 percent used 15IU, 33 percent used 10IU, 11.3 percent used 5IU, while as many as 10 percent used doses ranging between 30IU to 60IU.

**Table 6:** Use and dosage of oxytocin for various obstetric indications.

Dose	Max dose for stimulation/ augmentation of labor in primigravida Frequency (%)	Max dose for stimulation/ augmentation of labor in multipara Frequency (%)	Dose of oxytocin for prevention of PPH Frequency (%)
5IU	169 (24.0)	280 (39.8)	80 (11.3)
10IU	339 (48.1)	292 (41.4)	230 (32.6)
15IU	57 (8.1)	32 (4.5)	34 (4.8)
20IU	123 (17.4)	70 (9.9)	291 (41.4)
Others (30IU to 60IU)	17 (2.4)	31 (4.4)	70 (9.9)

Table 6 shows the different doses of oxytocin used by respondents for specific indicators.

Table 7 shows that the majority of respondents in both public (41.4%) and private (52.3%) health facilities use a maximum dose of 10IU of oxytocin for stimulation/augmentation of labor in a primigravida. Similar responses were also noted for the same indication in the multipara in both public (41.4%) and private (41.1%) health facilities. The majority use a maximum dose of 20IU units of oxytocin for the prevention of PPH in both public (43.2%) and private (40.0%) health facilities.

**Table 7:** Use and dosage of oxytocin for various obstetric indications according to respondent’s Sector of practice.

Dose	Max dose for stimulation/ augmentation of labor in primigravida Frequency (%)	Max dose for stimulation/ augmentation of labor in multipara Frequency (%)	Dose of oxytocin for prevention of PPH Frequency (%)
***Public Sector n = 267***			
5IU	78 (29.3)	106 (39.8)	30 (11.3)
10IU	110 (41.4)	110 (41.4)	82 (30.8)
15IU	28 (10.5)	10 (3.8)	14 (5.3)
20IU	47 (17.3)	32 (12.0)	115 (43.2)
Others	4 (1.5)	7 (2.6)	26 (9.4)
***Private Sector n = 438***			
5IU	90 (20.5)	172 (39.3)	49 (11.2)
10IU	229 (52.3)	180 (41.1)	146 (33.3)
15IU	32 (7.3)	21 (4.8)	18 (4.1)
20IU	73 (16.7)	38 (8.7)	175 (40.0)
Others	14 (3.2)	27 (6.1)	50 (11.4)

Twelve popular brands of oxytocin were assessed in this survey. These brands were previously audited for quality [2]. Respondents’ perception of the effectiveness and ineffectiveness of these brands vary significantly. These brands were de-identified for the purpose of this research. Table 8 gives the summary of the perception of effectiveness/ineffectiveness of these brands.

**Table 8:** Experience of quality of oxytocin brands used in obstetrics practice (*n* = 705).

Brands de-identified	Perceived quality of the different oxytocin brands		
	Effective Frequency (%)	Ineffective Frequency (%)	Don’t know Frequency (%)
A	450 (63.8)	29 (4.1)	226 (32.1)
B	430 (61.0)	17 (2.4)	258 (36.6)
C	122 (17.3)	24 (3.4)	559 (79.3)
D	602 (85.4)	38 (5.4)	65 (9.2)
E	149 (21.2)	33 (4.6)	523 (74.2)
F	65 (9.2)	22 (3.1)	618 (87.7)
G	38 (5.5)	26 (3.6)	641 (90.9)
H	48 (6.8)	17 (2.4)	640 (90.8)
I	50 (7.1)	19 (2.7)	636 (90.2)
J	31 (4.4)	18 (2.5)	656 (93.1)
K	23 (3.3)	16 (2.2)	666 (94.5)
L	38 (5.4)	0 (0.0)	667 (94.6)

Table 9 shows the pooled estimate of the effectiveness and ineffectiveness of the oxytocin brands used by the respondents. Overall, 98.3 percent have had experiences of effectiveness with the oxytocin brands, while 12.6 percent perceived that the oxytocin brands they used were ineffective, as seen in Table 9.

**Table 9:** Overall experience of effectiveness and ineffectiveness of oxytocin brands used by respondents.

Questions	Response	Frequency (%)
Perception of oxytocin quality	Effective	693 (98.3)*
	Ineffective	89 (12.6)*

* Multiple responses

The majority (64.3%) of respondents have no available means in place within their facility to document and/or report experience of ineffectiveness. Of the few who do, most (61%) document it in the patient’s case note, 27 percent in the clinical summary, and 12 percent in the pharmacovigilance form. In the event of oxytocin failure, 57 percent will resort to caesarean section, while 45.2 percent will change to another medicine, mainly misoprostol (40.1%). These results are summarized in Table 10.

**Table 10:** Practice following oxytocin use in obstetrics.

Question	Response	Frequency (%)
Availability of means of documenting/ reporting perceived oxytocin ineffectiveness	Available	252 (35.7)
	Not available	453 (64.3)
Reporting/documentation of perceived poor quality of medicines (n = 252)	Case note	154 (61.1)
	Clinical summary	68 (26.9)
	Pharmacovigilance form	30 (11.9)
Actions taken by respondents when the maximum recommended dose of oxytocin fails	Doubling the dose	37 (5.2)*
	Change the medicine	319 (45.2)*
	Caesarean section	402 (57.0)*

* Multiple responses

Table 11 shows that respondents in the public sector and doctors had significantly better knowledge of oxytocin storage (*p*<0.001).

**Table 11:** Factors affecting knowledge of proper storage of oxytocin.

Question	Response	Proper storage of oxytocin				
		Yes Frequency (%)	No Frequency (%)	Total	χ^2^	P
Sector of practice	Government	183 (68.5)	84 (31.5)	267	757.88	<0.001
	Private	176 (40.2)	262 (59.8)	438		
Cadre of health worker	Doctor	159 (57.8)	116 (42.2)	275	713.34	<0.001

## Discussion

Healthcare systems in most low-income countries are weak. This situation is further exacerbated when poor-quality medicines are in circulation. Our findings suggest poor knowledge of oxytocin storage among respondents. There was also inappropriate and inconsistent use of oxytocin, with the experience of ineffectiveness of oxytocin brands used among respondents. Oxytocin is a peptide with a highly unstable structure. The biggest obstacle to oxytocin quality is storage and handling before patient use. The storage condition of oxytocin has been widely reported as inappropriate [18]. Oxytocin is a heat-sensitive medicine and should be kept between 2°C and 8°C. A previous study in India documented that most physicians and nurses did not know how oxytocin should be stored [15], while an assessment in Nepal found that only 8.6 percent of health facilities stored oxytocin in the refrigerator [19]. Our study showed that only 52 percent of respondents knew that oxytocin should be stored in the refrigerator, although we did not assess the actual practice—which may be much lower. A further assessment of the association between good knowledge of proper storage of oxytocin with sector of respondents’ practice revealed that about 68 percent of healthcare providers in the public sector and 40 percent in the private sector knew oxytocin should be stored in the refrigerator. This difference was statistically significant with *p*<0.001. There was also a statistically significant difference between cadre of staff and knowledge *p*<0.001. As many as 41 percent of doctors and 52 percent of nurses did not know that oxytocin should be stored in the refrigerator. It is also very important to note other factors that could affect quality of oxytocin are outside the control of the healthcare provider, including include difficulty of a procurement agency to identify high-quality products [8] and poor supply chain and logistics management (e.g., inadequate transport and storage facilities along the supply chain and lack of stable electricity) [11]. The oxytocin vials lack temperature–time indicators as seen in vaccine vials to enable healthcare providers to ensure quality at the point of use [7].

According to the summary product characteristics of oxytocin, the therapeutic dose for induction, stimulation, and augmentation of labor for a medically recommended reason—including prevention of PPH—is 5IU [4]. However, WHO recommends 10IU (IV/IM) for prevention and treatment of PPH [2]. Most evidence-based guidelines (U.K. and Canada) recommend a low dose of oxytocin for induction and augmentation [20]. Our findings revealed that different doses of oxytocin (low and high) were used by healthcare providers in this study even within the same facility. A very high proportion of the respondents in our study used doses beyond the maximum recommended for intrapartum use in primigravida and in multiparas (who obviously need lower doses). About 41 percent of respondents used double the WHO-recommended dose. Nearly 10 percent used doses ranging from 30IU to 60IU of oxytocin, which translates to use of two to six vials for a 10IU vial to achieve the desired uterine contraction. This may just be an indication of failed quality, supporting the report in literature that healthcare providers in Africa often used up to three vials to get the desired effect of one [14]. The findings in our study are similar to reports from a previous study in Karnataka, India [15]. This encourages waste and diverts limited resources from saving the lives of other women and improving maternal health. It also increases the clients’ healthcare spending.

In assessing respondents’ perceived effectiveness/ineffectiveness of oxytocin used in their practice, up to 13 percent have experienced use of an ineffective brand of oxytocin at one time or the other. Lack of suspicion of medicines quality by healthcare providers as a possible cause of disease progression or contributor to treatment outcome may have influenced this level of perceived ineffectiveness: medicinal products are supposed to protect patients and save lives so should be 100-percent effective. The findings correlate with reports from laboratory assays of the high prevalence of poor-quality oxytocin samples in low- and middle-income countries [11,21]. No previous study within our search of published literature had assessed healthcare provider perceived effectiveness or ineffectiveness of oxytocin used in their clinical practice, which posed a challenge in making comparisons.

The high level of knowledge of the correct definition of PPH is not surprising, as our respondents were supposedly highly skilled healthcare providers. Similarly in Ethiopia, 82.4 percent of skilled healthcare providers defined PPH correctly [22].

The pattern of indications for oxytocin use is similar to the Nepal study in which the majority (78%) of health service providers used oxytocin for prevention and management of PPH, while 59 percent used oxytocin for augmentation and induction of labor [19]. Our study is consistent with previous studies that oxytocin may be very commonly and inappropriately used for induction and augmentation of labor [20,23], evident that a significant proportion (80%) almost routinely use oxytocin in augmentation of labor [5,24,25].

It was noted that the majority (57%) of respondents performed a caesarean section when the maximum recommended dose of oxytocin failed, while 5.2 percent doubled the dose of oxytocin used. Though rare, overdose following the doubling or tripling of a potent oxytocin injection could lead to uterine hypertonicity, spasm, tetanic contraction, or rupture of the uterus [26]. Cervical or vaginal laceration, uteroplacental perfusion, and water intoxication with seizures following antidiuretic effect of oxytocin (including death) have been documented with severe toxicity [26]. Possible consequence of poor oxytocin quality as reported in previous studies could result in excessive and inappropriate use of oxytocin, and performing unnecessary surgical procedures could lead to avoidable complications and even death [13,14,20]. Despite these experiences, only about 36 percent of respondents had a system in place for documenting or reporting perceived ineffectiveness of drugs used. This further supports reports that healthcare providers often do not suspect or document drug quality used in the course of practice [13].

It is possible that the inappropriate and inconsistent use of oxytocin—especially overdosing—likely led to the spuriously high perception of medicine effectiveness among respondents. This is coupled with the lack of suspicion of medicine ineffectiveness by clinicians as a possible root cause of poor treatment response or disease progression. Poor knowledge of oxytocin storage and consequent poor storage practices could have contributed to the ineffectiveness reported by some respondents.

### Strengths and limitations

There is a dearth of published data on the perceived quality of oxytocin used by healthcare providers. This study contributed to the much-needed data on this topical issue, especially in low- and middle-income countries with high maternal mortality due mainly to hemorrhage. The representativeness of the respondents from the public and private sectors and the involvement of all levels of the health system (tertiary/secondary/primary) across Lagos State is a major strength of this study. However, the study did not include middle- and lower-level healthcare providers (e.g., community health officers, community health extension workers, traditional birth attendants) who also use oxytocin in their practice even though they are not approved to use it at that level. There could also be the issue of possible recall bias, since the responses were self-reported. A qualitative aspect to complement the quantitative data collected will be considered in further studies.

## Conclusion and recommendation

This study brings to the consciousness of healthcare providers in Nigeria the possible contribution of poor medicines quality to the poor maternal health risks and indices in Nigeria. It further highlighted the level of pharmacovigilance in the healthcare system and, by extrapolation, in various similar settings in other low and middle-income countries. Other findings include the not-so-encouraging level of knowledge about proper storage conditions, consequent storage practice for oxytocin, and by extension poor clinical outcomes of poor-quality oxytocin. These have dire consequences that span from the impact on the overall health of the patient to the death of the patient and loss of confidence in the health system.

Over half of respondents will resort to surgical procedures when the administered oxytocin is ineffective. This calls for an urgent plan to put in place a standard protocol to guide practices in the storage and use of oxytocin. Proper reporting channels on suspected poor quality of drugs should be improved, including continued education of health workers on the use of pharmacovigilance forms and ensuring availability. There is a need for continuous and expanded preservice and in-service training of healthcare providers to develop skills in drug safety monitoring, including the suspicion of drug quality in the chain of events that could possibly result in poor health outcomes. There is also a need to ensure good oxytocin manufacturing quality and to strengthen oxytocin supply chain management, including procurement, transport, and storage while providing stable electricity, as these factors are outside the complete control of the healthcare provider.

## Supporting information

S1 File Data set. (XLS)

S2 File Questionnaire. (DOC)
