# Supplementary material for: Clinical experiences with the use of oxytocin injection by healthcare providers in a southwestern state of Nigeria: A cross-sectional study
Source: PLoS One. 2019 Oct 10;14(10):e0208367. doi: 10.1371/journal.pone.0208367 (PMC6786624; doi:10.1371/journal.pone.0208367)
Supplement: S2 File — (DOC) [file pone.0208367.s002.doc]

**RESPONDENT’S INFORMED CONSENT FORM**

**HREC ASSIGNED No: ADM/DCST/HREC/APP/1800**

**Title of Project:** Clinical experiences of Oxytocin injection quality used by healthcare providers in Lagos State

**Name & Affiliation of Researcher:** The Principal Investigator for this project is Dr. Chioma Ejekam of the Department of Community Health, Lagos University Teaching Hospital in partnership with other co-investigators in the teaching hospital.

**Introduction:**

Post-partum haemorrhage (PPH) is a leading cause of maternal mortality in Nigeria and most low-income countries and it is preventable. The World Health Organization(WHO) has recommended Oxytocin an effective and the safest drug of first choice in the prevention and treatment of PPH in the active management of the third stage of labour for improving the health care of women during childbirth. However, there are concerns about its quality.

**Purpose(s) of the research:** This study hopes to assess the Clinical experience of healthcare providers in Lagos State with the quality of oxytocin often used in their practice.

**Procedure of the research:** The questionnaire is self-administered and will obtain information on your occupational history, knowledge of oxytocin and clinical experiences with the quality of oxytocin often used in your practice. To fill this questionnaire takes about 7-10minutes.

**Potential benefit(s):** The study may unveil the possible contribution of poor quality oxytocin to the maternal morbidity and mortality statistics in Nigeria and ultimately improve patient’s safety. There is no monetary benefit from participation.

**Potential Risks:** The study is a questionnaire-based survey and the respondents are not considered a vulnerable population hence no harm is anticipated.

**Confidentiality:** All information obtained in this study will be given code numbers and no name will be recorded. This cannot be linked to you in any way and your name or any identifier will not be used in any publication or reports from this study.

**Willingness to Participate:** Your participation in this study is entirely voluntary. You have the right to decide to participate or not. The data obtained does not constitute part of your employment records.

**What Happens to Research Participants and Communities When the Research is over?**

The researcher will inform you of the outcome of the research through a medical journal. There is no conflict of interest whatsoever on the part of the author.

**Statement of Person Giving Consent:**

I have read the description of the research. I understand that my participation is voluntary. I know enough about the purpose, methods, risks and benefits of the research study to judge that I want to take part in it. I understand that I may freely stop being part of this study at any time.

Signature: ……………………………………………. Date: ………………………………….

**For further enquiry, please contact:**

Researcher

Dr C.Ejekam

Email contact: bfchioma@yahoo.com

Dept. of Community Health, Lagos University Teaching Hospital

Lagos University Teaching Hospital (LUTH)

Health Research and Ethics Committee, Administrative Building, Room 107,

Lagos University Teaching Hospital, Idi-Araba, Lagos

**CLINICAL EXPERIENCES OF OXYTOCIN INJECTION QUALITY USED BY HEALTH CARE PROVIDERS IN LAGOS STATE**

**SECTION A: SOCIO-DEMOGRAPHICS OF RESPONDENTS**

1. Age at last birthday………..
2. Gender A: Male ( ) B. female ( )

**SECTIONB: OCCUPATIONAL HISTORY OF RESPONDENTS**

1. Cadre a) doctor ( ) b) Nurse/Midwives
2. Years of working experience…………
3. What type of health facility do you practice in? a) Public ( ) b) Private ( )
4. Have you been trained in the use of Oxytocin? a) Yes ( ) b) No ( )

**SECTION C: GENERAL KNOWLEDGE**

1. What is postpartum haemorrhage?
2. Blood loss less than 200ml b) Blood loss less than 400ml c) Blood loss less

than 500ml d) Blood loss 500ml and above e) Others………..

f) Don’t know

1. What will you assess for in a woman with postpartum haemorrhage?
2. vital signs (BP, Pallor, temperature) (b) Uterine tone

c) Haemoglobin level (d) Others……….. (e) Don’t know

1. What do you use oxytocin for? (you may tick more than one answer)

a) stimulation of labour b) augmentation of labour

c) Induction of labour d) management of post-partum Hemorrhage

e) Others, please specify ……

1. Which cadre of health care provider administers oxytocin in your practice?
2. Doctors b) nurse/midwives c) both doctors and nurses/midwives
3. Who often procures the oxytocin? a) Clients b) Hospital facilities
4. How is oxytocin to be stored? a) in refrigerator b) on the drug shelve c) in the dark

d) others ………………

**SECTION D: CLINICAL EXPERIENCE WITH OXYTOCIN**

1. What is the maximum dose of oxytocin that you use for the

Stimulation/augmentation/induction of labour in primiparous women?

5 IU b) 10 IU c) 15 IU d)20 IU e) Others

1. What is the maximum dose of oxytocin that you use for the

stimulation/augmentation/induction of labour in multiparous women?

5 IU b) 10 IU c) 15 IU d) 20 IU e) Others

1. What dose of oxytocin do you use for the prevention of primary postpartum hemorrhage?
   1. 5 IU b) 10 IU c) 15 IU d) 20 IU e) Other
2. What do you monitor to ensure that the oxytocin is effective when used in labour?
   1. at least three uterine contractions of 40 to 60 seconds each in over 10 minutes
   2. cervical dilatation c) both a. and b. d) none of a. and b. e) Others……….
3. What are your experiences with these brands mentioned below (Tick as appropriate for each

brand)

| **S/N** | **Oxytocin brand** | **Effective** | **Ineffective** | **Do not know/have not used it** |
| --- | --- | --- | --- | --- |
| a) | De-identified-A |  |  |  |
| b) | De-identified-B |  |  |  |
| c) | De-identified-C |  |  |  |
| d) | De-identified-D |  |  |  |
| e) | De-identified-E |  |  |  |
| f) | De-identified-F |  |  |  |
| g) | De-identified-G |  |  |  |
| h) | De-identified-H |  |  |  |
| i) | De-identified-I |  |  |  |
| j) | De-identified-J |  |  |  |
| k) | De-identified-K |  |  |  |
| l) | De-identified-L |  |  |  |
| E) | Others…………….. |  |  |  |

1. Is there a process/system (e.g. clinical summary, case note or a form) in place to document or

report perceived ineffectiveness of oxytocin or drugs used in your practice? a) Yes b) No

1. If ‘yes’ to question 18, please specify where you document or report this?

a) Case note b) Clinical Summary c) Pharmacovigilance form

20. What do you do when maximum recommended dose of oxytocin fails?

a) double the dose b.) change drug c.) Caesarean section d.) Others ……

21. If you change drugs, which drug do you change to?

a) Ergometrine b.) Misoprostol c.) Dinoprostone d.) Carbetocin e) Carboprost

22. Please, outline the consequences or implications of poor oxytocin quality that may you have

seen or experienced in your clinical practice.

…………………….

…………………….

……………………

23. What are the side-effects you have suspected may be as a result of oxytocin use?

a) Confusion b.) seizures c.) difficulty in breathing d.) dizziness e.) palpitations

f.) headache (continuing or severe) g.) hives h.) pelvic or abdominal pain (severe)

i.) skin rashes or itching
